# Ionic Liquids Effect on the Stability of 17-Electron Cation Product of the Electrochemical Oxidation of Cymantrene

**DOI:** 10.3390/molecules27217428

**Published:** 2022-11-01

**Authors:** Angel A. J. Torriero

**Affiliations:** School of Life and Environmental Sciences, Deakin University, Burwood, Victoria 3125, Australia; angel.torriero@deakin.edu.au; Tel.: +61-3-9244 6897

**Keywords:** cymantrene, ionic liquids, cyclic voltammetry, Lewis base

## Abstract

The oxidative electrochemistry of cymantrene, CpMn(CO)_3_ (**1**; Cp = [η^5^-C_5_H_5_]^–^), was examined in ionic liquids (ILs) composed of anions of varying Lewis base properties. It was observed that the cyclic voltammetric responses strongly depended on the nucleophilic properties of the IL anion. Still, all observations are consistent with the initial formation of **1^+^** followed by an attack from the IL anion. In bis(trifluoromethylsulfonyl)amide [NTf_2_]-based ILs, the process shows close to ideal electrochemical reversibility as the reaction between **1^+^** and [NTf_2_] anion is very slow. On the other hand, in tetrafluoroborate and trifluoromethanesulfonate-based IL, the oxidation of **1** shows different levels of electrochemical reversibility with a marked sign of anion attack to **1^+^**. In contrast, **1** exhibits an irreversible oxidation process in hexafluorophosphate-based IL. The reaction rate constants for the interaction of **1^+^** with the different IL anions were estimated by fitting the experimental data to digital simulations of the proposed mechanism. Besides, the use of [NTf_2_]-based ILs as a supporting electrolyte in CH_2_Cl_2_ was also examined. The oxidation process of **1** shows a close to ideal electrochemical reversibility but low to non-chemical reversibility. This study illustrates the wide range of electrochemical environments available with ILs and demonstrates their limited utility for investigating the redox properties of metal carbonyl compounds. It also intends to warn the reader on how the IL media may influence an electrochemical study if care is not exercised.

## 1. Introduction

The solvent selection is one of the essential features for the success of a planned reaction, as the solvent choice can affect equilibrium and rate-determining reaction times, as well as the product distribution [1,2]. In molecular solvents, the solvent-solute interaction may involve dipole–dipole interactions and other intermolecular forces such as hydrogen bonding, π-interactions, London dispersion forces, or even coordinative bonds. Since ionic liquids, ILs, have properties completely different from molecular solvents (e.g., water and organic solvents), it is likely that they will have dramatic effects on reactions occurring within them [3,4,5,6]. However, the structural origins that control ILs physicochemical properties are still unclear. Considering the number of already available ionic liquids, chemists and electrochemists need, in addition to their intuition, some general rules to facilitate the choice for a specific electrochemical application. Accordingly, there is an obvious need to characterise the nucleophilicity of ILs. While studies on the role of molecular solvents and/or electrolytes in organometallic redox systems have been widely studied for many years [7,8,9], it is perhaps surprising that analogous studies in IL media have not been equally reported.

To probe the effect of IL anions on electrogenerated species, the redox chemistry of the piano-stool complex MnCp(CO)_3_ (**1**, Cp = [η^5^-C_5_H_5_]^–^), often referred to as cymantrene, will be investigated in a range of ILs as the solvent and in dichloromethane (CH_2_Cl_2_) having the ILs as supporting electrolyte. This complex was selected because its electron transfer and coupled chemical reaction pathways have been well-characterised in conventional media and vary substantially with the nature of the molecular solvent and nucleophile used [10,11,12,13,14,15,16,17,18,19]. It is known that the electrophilicity of the 17-electron organometallic radical (**1^+^**), generated as the oxidation product of 18-electron cymantrene (**1**, Equation (1)), is susceptible to cleavage reactions with either donor solvents, supporting electrolyte anions, or added nucleophiles (L in Equation (2)) [18], undergoing fast substitutions of one or two carbonyls (CO) ligands. The chemical reaction that follows the electron transfer may be expected as removing an electron from **1** would weaken the Mn-CO bonds (owing to reduced metal-to-CO back-bonding) and perhaps the Mn-Cp bond [11]. In contrast, in weekly coordinating media, such as CH_2_Cl_2_ containing tetrabutylammonium tetrakis (pentafluorophenyl)borate as the supporting electrolyte, only the chemically reversible one-electron oxidation process (Equation (1)) was observed [11].


(1)

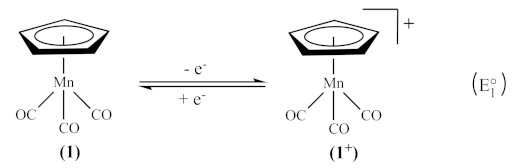




(2)

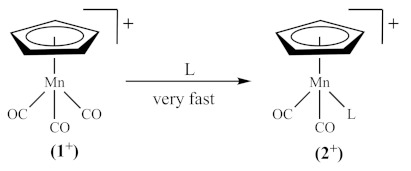



The susceptibility mentioned above of **1^+^** to nucleophilic attack by the solvent or supporting electrolyte makes the electrochemical oxidation of **1** an excellent system to examine the chemical reactivity of the IL media. This work describes the oxidative electrochemistry of **1** in five different ILs. The anions employed in these studies, hexafluorophosphate, [PF_6_]^–^, tetrafluoroborate, [BF_4_]^–^, bis-(trifluoromethylsulfonyl)amide, [NTf_2_]^–^, and trifluoromethanesulfonate, [CF_3_SO_3_]^–^, cover an important range of Lewis basicity and, as will be described, exhibiting markedly different reactivity toward electron-deficient species. The oxidative electrochemical behaviour can be explained by the initial generation of **1^+^** and its subsequent reactivity toward the IL anion. Besides, the oxidation of **1** was also examined in CH_2_Cl_2_ with IL or tetrabutylammonium hexafluorophosphate, [Bu_4_N][PF_6_], added to serve as the supporting electrolyte. The present study, therefore, pretends to demonstrate the substantial range of electrochemical environments available with ILs and exemplifies the importance of the reactivities of the IL anions in electrochemical studies performed in IL media.

## 2. Results and Discussion

Ionic liquids are classified according to the Lewis acidity or basicity of their component cations and anions, respectively [20]. Based on the basicity, the four anions employed in this work [NTf_2_]^–^, [PF_6_]^–^, [BF_4_]^–^, [OTf]^–^ were initially classified into a “neutral” category as they were considered extremely weak base anions [20], or more generally as “non-coordinating anion” [21,22]. However, it has recently been recognised that grouping these anions into that category may be misleading since they are Lewis bases, and their interaction with Lewis acid centres is well reported [23,24,25]. A summary of the electrochemical data for oxidation of **1** in each of the ILs is presented in Table 1.

Trifluoromethanesulfonate ([CF_3_SO_3_]^–^; [OTf]^–^ or triflate) possess a relatively moderate coordinating ability to metal centres similar to perchlorate and allegedly stronger than [PF_6_] and [BF4] anions, with many triflate-containing complexes been previously isolated and characterised [21,25,26]. This anion, therefore, may provide ILs with coordination properties similar to solvents such as acetonitrile.

**Figure 1:**
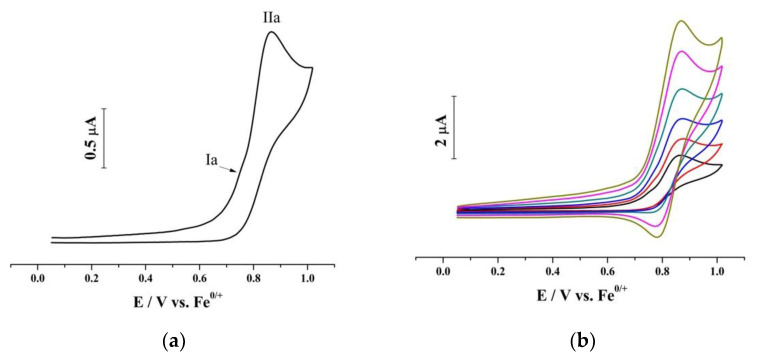
Cyclic voltammograms obtained for 5 mM **1** in [emim][OTf] at a glassy carbon electrode (d = 1 mm). (**a**) over a wide potential range with a scan rate of 0.02 V s^−1^ and (**b**) over the potential region where the oxidation occurs at scan rates of 0.02, 0.05, 0.10, 0.20, 0.40, and 0.60 V s^−1^. T = 21 ± 1 °C.

Figure 1 shows the cyclic voltammogram of **1** in [emim][OTf] obtained at a scan rate of 0.02 V s^−1^. Two processes are observed: an irreversible oxidation at 0.869 V vs. Fc^0/+^ (indicated as peak IIa) and a shoulder or pre-peak at ca. 0.724 V vs. Fc^0/+^ (peak Ia). When the potential is scanned in the cathodic direction, no new processes are observed. Since the cyclic voltammetry resembles that previously reported in acetonitrile (0.1 M [Bu_4_N][ClO_4_]) containing triphenylphosphine as the nucleophile [19]; by analogy, we assign processes IIa to the oxidation of **2** to **2^+^** and the pre-peak Ia to the rapid CO substitution in **1^+^** to give the cation **2^+^** (Equation (2)). It is noteworthy that the potential separation between the two oxidation processes is smaller than that previously reported in acetonitrile [19].

**Scheme 1:**
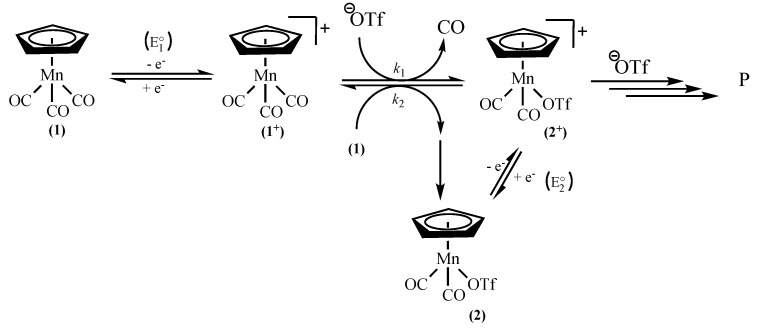
Catalytic $\vec{E}C\overset{\leftarrow }{E}C$ pathways for the oxidation of **1** in [emim][OTf].

An additional feature accompanies the oxidation of **1** in [emim][OTf]. Process IIa is unstable to further attack by the coordinating solvent, appearing as an irreversible process when the voltammograms are recorded at slow scan rates (≤0.1 V s^−1^). This observation supports the reaction scheme outlined below (Scheme 1), which involves a catalytic substitution of carbonyl by [OTf]^–^. The initially formed **1^+^** is rapidly attacked by [OTf]^–^ to create **2^+^**, which, in turn, reacts with **1** following a disproportionation-type reaction to produce **2** and **1^+^**. The ligand-substitution mechanism is completed by back electron transfer at the electrode potential $\overset{\leftarrow }{E_{2}^{0}}$. The voltammetric results confirmed that the redox potential of **2^0/+^** (named as $E_{2}^{0}\;$ in Scheme 1) is more positive than the redox potential of **1^0/+^** ($E_{1}^{0}$). Besides, a followed-up reaction between **2^+^** and [OTf]^–^ may account for the irreversibility observed at low scan rates.

**Figure 2:**
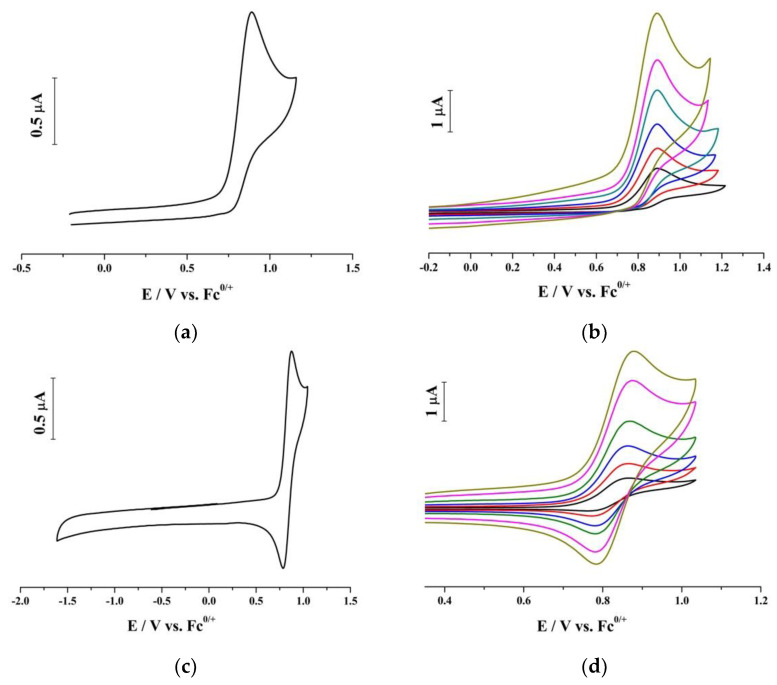
Cyclic voltammograms obtained for 5 mM **1** in (**a**,**b**) [bmim][PF_6_], (**c**,**d**) [bmim][BF_4_] at a glassy carbon electrode (d = 1 mm). (**a**–**c**) over a wide potential range with a scan rate of 0.1 V s^−1^ and (**b**–**d**) over the potential region where the oxidation occurs at scan rates of 0.02, 0.05, 0.10, 0.20, 0.40, and 0.60 V s^−1^. T = 21 ± 1 °C.

Hexafluorophosphate has been widely employed as a supporting electrolyte anion in electrochemical work because of its very low Lewis basicity. However, it may not be entirely innocent since there is evidence that it can coordinate with or act as a nucleophile toward highly electron-deficient species [18,27,28]. This aspect of its reactivity may be expected to be enhanced in IL media since the [PF_6_]^–^ concentration in neat IL is roughly a factor of 50 greater than when it is employed as a supporting electrolyte in organic solvents, typically at a concentration of 0.10 M. Indeed, the results indicate that [PF_6_]-based ILs have considerable reactivity toward electron-deficient species. Figure 2a,b show the cyclic voltammetric response of the oxidation of **1** in [bmim][PF_6_] obtained at scan rates between 0.02 and 0.60 V s^−1^. The voltammograms show the fully irreversible oxidation of **1** at 0.891 V vs. Fc^0/+^, which is maintained even when scan rates of 10 Vs^−1^ are used. This effect may be attributed to the attack by [PF_6_]^–^ on **1^+^** in a reaction analogous to that proposed for triflate in Scheme 1. However, the reaction rate of the followed-up reaction between **2^+^** and [PF_6_]^–^ is larger than that observed in [emim][OTf], which accounts for the irreversibility observed at all the scan rates used.

Tetrafluoroborate has also been widely employed as a supporting electrolyte anion in electrochemical work because of its low Lewis basicity. However, numerous crystal structures show [BF_4_]^–^ as a monodentate or bidentate bridging ligand [21,23]. Furthermore, it has also been realised that this anion may act as a nucleophilic fluoride source [29,30]. Based on this, it is possible to arrive at the early conclusion that [BF_4_]^–^ could be more reactive than [PF_6_]^–^. Nevertheless, despite the cyclic voltammetric response of the oxidation of **1** in [bmim][BF_4_], obtained at scan rates between 0.02 and 0.60 V s^−1^, shows measurable reactivity toward **1^+^** (Figure 2d), they reflect a more reversible behaviour than that seen in [bmim][PF_6_] (Figure 2) and [emim][OTf] (Figure 1). Figure 2c,d, show the cyclic voltammetric response of the oxidation of **1** in [bmim][BF_4_] obtained at scan rates between 0.02 and 0.60 V s^−1^. These voltammograms (see also Table 1) reveal a decrease in peak current ratios at lower scan rates, which may be attributed to a relatively slow attack by [BF_4_]^–^ or fluoride on **1^+^** in a reaction analogous to that proposed for triflate in Scheme 1. However, no clear indication of two oxidation waves is observed, which may imply that $E_{1}^{0}≅E_{2}^{0}$.

**Figure 3:**
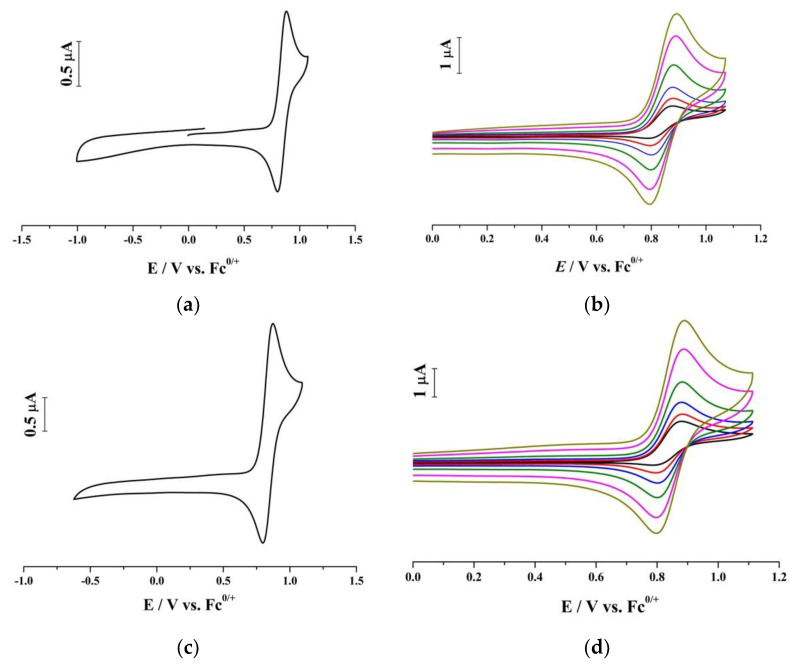
Cyclic voltammograms obtained for 5 mM **1** in (**a**,**b**) [bmpyr][NTf_2_], (**c**,**d**) [emim][NTf_2_] at a glassy carbon electrode (d = 1 mm). (**a**–**c**) over a wide potential range with a scan rate of 0.1 V s^−1^ and (**b**–**d**) over the potential region where the oxidation occurs at scan rates of 0.02, 0.05, 0.10, 0.20, 0.40, and 0.60 V s^−1^. T = 21 ± 1 °C.

The oxidation of **1** shows even more significant electrochemical reversibility when performed in [NTf_2_]-based ILs (Figure 3). This comment is supported by (*i*) the linear behaviour of the oxidative peak current $I_{p}^{ox}$ of **1** in [bmpyr][NTf_2_] and [emim][NTf_2_] vs. the square root of scan rate over the range 0.1 to 1.00 V s^−1^, and with concentrations over the range 2.5–10 mM; and (*ii*) the ∆*E*_p_ ≈ 70 mV, commonly observed for electrochemically reversible processes in ILs [31,32]. Nevertheless, despite the high electrochemical reversibility of the **1^0/+^** couple in these ILs (Table 1) and the absence of new cathodic voltammetric peaks appearing after the oxidation of **1**, there is still evidence of a slow homogeneous reaction coupled to the electron transfer. The $I_{p}^{red}/I_{p}^{ox}$ ratio obtained in these two ILs attain unity at scan rates ≥1 V s^−1^, deviating from unity as the scan rates decrease. Such an interaction is consistent with the previously reported interaction of [NTf_2_]-based ILs with [CpFe(CO)_2_]_2_^+^ [3].

**Figure 4:**
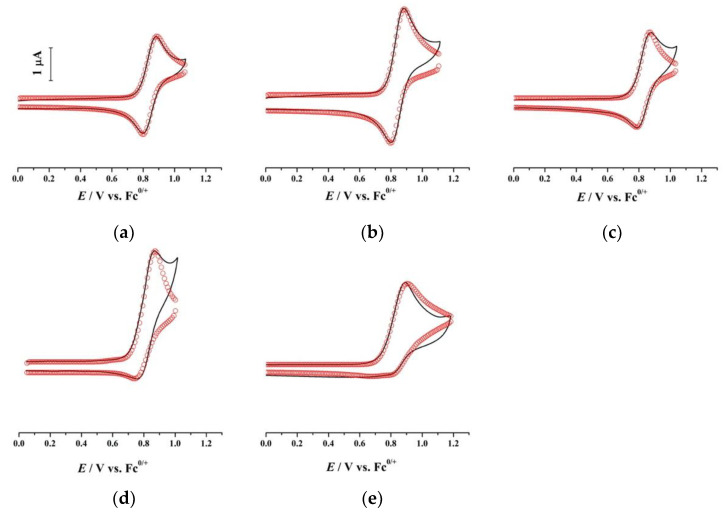
Comparison of experimental (solid lines) and simulated (open circles) cyclic voltammograms for the oxidation of 5 mM **1** in (**a**) [bmpyr][NTf_2_], (**b**) [emim][NTf_2_], (**c**) [bmim][BF_4_], (**d**) [emim][OTf], and (**e**) [bmim][PF_6_]. The mechanism depicted in Scheme 1 and data from Table 1 were used for simulations. Scan rate = 0.20 Vs^−1^. T = 294 K, electrode area = 0.7 mm^2^, charge-transfer coefficient = 0.5, heterogeneous standard rate constant = 1.0 × 10^−2^ cm s^−1^, double-layer capacitance = 0.60 μF.

While [NTf_2_] anion can act as a nucleophile toward highly electron-deficient metal carbonyl species (e.g., CpFe(CO)_2_(NTf_2_)) [9], the kinetics of this reaction is very slow in ILs. Therefore, rate constants for the IL anion’s attack on **1** were calculated by fitting experimental voltammetric responses to digital simulations of the process shown in Scheme 1 to measure the relative reactivity of the anions used in this investigation (Figure 4). The value of *k*_2_ was first arbitrarily chosen, and the value of *k*_1_ changed. When the *k*_1_ listed in Table 1 is used, the simulations exhibit excellent agreement with experimental results. It is worth mentioning that the mechanism is not critically dependent on the value of *k*_2_ if it is chosen over the range 4 < *k*_2_ > 30 for all the scan rates used in this work. Significantly, the oxidation of **1** in [NTf_2_]-based ILs (Figure 4a,b) shows the smaller *k*_1_ values, which is consistent with observation performed with the use of [(Cp)Fe(CO)_2_]_2_ as the probe molecule [3]. Nevertheless, the reactivity toward **1^+^** appears to be a usefully and more sensitive method of discriminating the relative nucleophilicity of IL anions when compared with [(Cp)Fe(CO)_2_]_2_. Moreover, simulations of the more complex chemistry in the [PF_6_]-based IL also gave excellent agreement with the experimental voltammograms over a wide range of scan rates when *k*_1_ = 1.8 × 10^4^ s^−1^ and *k*_2_ = 1 × 10^−3^ s^−1^, is used to simulate the irreversible conversion of **2^+^** to a product.

**Figure 5:**
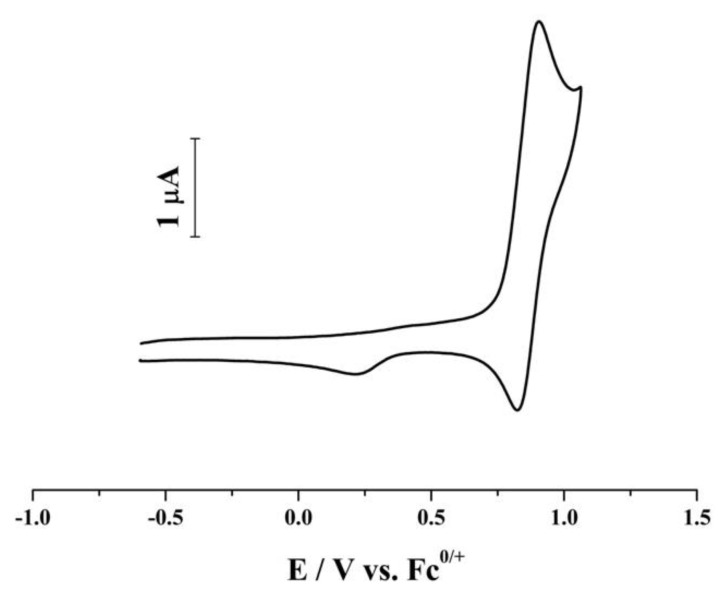
Cyclic voltammograms obtained for 5 mM **1** in [bmim][BF_4_] containing 200 ± 10 ppm water at a glassy carbon electrode (d = 1 mm). Scan rate = 0.10 V s^−1^, T = 21 ± 1 °C.

Water is always present as an impurity in ILs and can compete with the IL anions for electrophilic radical cations. To support the contention that the chemical reactions coupled to **1^+^** observed in ILs is primarily due to the IL anion and not to adventitious water, the cyclic voltammetry of **1** in [bmim][BF_4_] was examined after the deliberate introduction of additional water. Under these conditions, a new irreversible reduction process coupled with the oxidation of **1** appears at ∼0.21 V vs. Fc^0/+^ (Figure 5). Based on Scheme 1, the formation of [CpMn(CO)_2_(H_2_O)] is expected as a result of the reaction between water and **1^+^**. Accordingly, this new peak is assigned to its one-electron reduction process.

**Figure 6:**
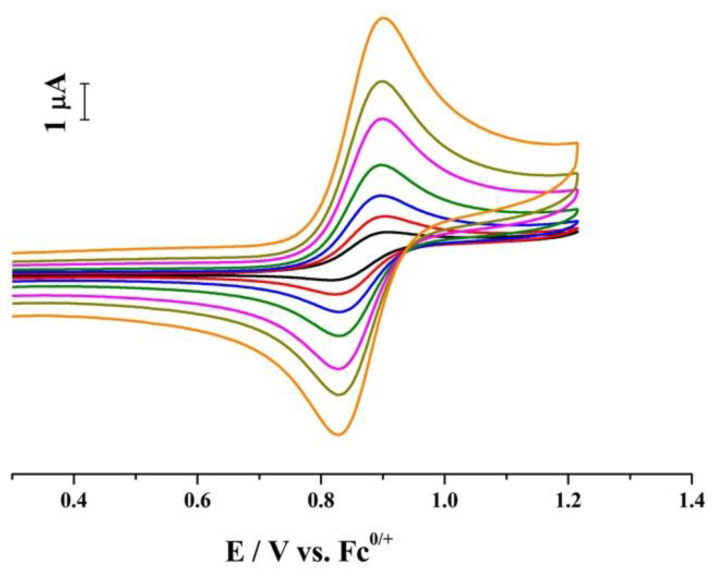
Cyclic voltammograms obtained for 1 mM **1** in CH_2_Cl_2_ containing 1 M [bmpyr][NTf_2_] as supporting electrolyte at a glassy carbon electrode (d = 1 mm) over the potential region where the oxidation occurs at scan rates of 0.02, 0.05, 0.10, 0.20, 0.40, 0.60, and 1.0 V s^−1^. T = 21 ± 1 °C.

Due to the [NTf_2_]^–^ low nucleophilicity, [emim][NTf_2_] and [bmpyr][NTf_2_] may be considered useful as weakly coordinating electrolytes in molecular solvents. To this end, the behaviour of [bmpyr][NTf_2_] as a supporting electrolyte in CH_2_Cl_2_ was tested. Oxidation of 1.0 mM **1** in CH_2_Cl_2_, containing 1 M [bmpyr][NTf_2_] as the supporting electrolyte, showed close to ideal electrochemical reversibility (Figure 6), with *E*_m_ = 0.864 V versus Fc^0/+^, peak current ratios increasing from 0.87 to 0.96 when the scan rate was increased from 0.1 to 1.0 V s^−1^, and ∆*E*_p_ = 66 mV at 0.1 Vs^−1^. Interestingly, the diffusion coefficient, extracted from the voltammetric analysis of **1** in CH_2_Cl_2_ containing 1 M [bmpyr][NTf_2_] as the supporting electrolyte, was 2.1 × 10^−6^ cm^2^ s^−1^, which is larger than 0.9 × 10^−6^ cm^2^ s^−1^, obtained for the oxidation of **1** in CH_2_Cl_2_ (1 M [Bu_4_N][PF_6_]). This may imply a lower viscosity of the solvent media when 1 M [bmpyr][NTf_2_] is used as the supporting electrolyte. Nevertheless, bulk electrolysis results show a lack of chemical reversibility. Oxidation of 1.4 mM **1** at 1 V vs. Fc^0/+^ in CH_2_Cl_2_ (0.5 M [bmpyr][NTf_2_]) makes the solution turn to a bluish-red colour during the first 4 min of electrolysis, which colour is characteristic of stable **1^+^**. Nevertheless, the colour disappears during the electrolysis. The total coulombs gave n_app_ = 4.0 electrons per molecule of **1**, indicating that ligand substitution takes place in this solvent system.

## 3. Materials and Methods

Reagents. High purity grade (>99.9%) 1-methyl-3-butylimidazolium hexafluorophosphate, [bmim][PF_6_], 1-methyl-3-butylimidazolium tetrafluoroborate, [bmim][BF_4_], 1-methyl-3-ethylimidazolium bis(trifluoromethylsulfonyl)amide, [emim][NTf_2_], 1-methyl-1-butylpyrrolidinium bis(trifluoromethylsulfonyl)amide, [bmpyr][NTf_2_], 1-methyl-3-ethylimidazolium trifluoromethanesulfonate, [emim][OTf], were purchased from Merck. Reagent-grade dichloromethane (CH_2_Cl_2_, Merck, Darmstadt, Germany), was distilled from appropriate drying agents and stored over CaH_2_ prior to use. Ferrocene (Fc, 98%, Sigma-Aldrich, Castle Hill, Australia), Silver trifluoromethanesulfonate (AgOTf, 97%, Sigma-Aldrich, Castle Hill, Australia) Tetrabutylammonium hexafluorophosphate ([Bu_4_N][PF_6_], ≥99%, Sigma-Aldrich, Castle Hill, Australia), and cyclopentadienylmanganese (I) tricarbonyl (**1**, 98%, Strem, Newburyport, MA, USA), were used as received from the manufacturer.

Apparatus and procedures. Experimental procedures were performed in a nitrogen-filled glovebox. Glassware used for electrochemical experiments was rinsed with nanopure water, dried at 120 °C for at least 12 h, and cooled under vacuum immediately before use. Unless otherwise stated, the water concentration in IL media was 80 ± 10 ppm, as determined with a Model 756 Karl Fischer Coulometer (Metrohm, Mitcham, Australia) using hydranal Coulomat AG as the titrant.

Voltammetric experiments were performed with an Interface1000 electrochemical workstation (Gamry, Warminster, PA, USA). Uncompensated resistance was measured in a potential region where no Faradaic reaction occurs, using the RC time constant method available with the instrument. This resistance was 85% compensated by the same instrument. The commercially available software package DigiElch (Gamry, Warminster, PA, USA) was used to simulate the voltammetric responses. The voltammograms were obtained using a conventional three-electrode arrangement, consisting of a 1.0 mm diameter glassy carbon (GC, effective area = 0.7 mm^2^) disk (ALS, Tokyo, Japan), a platinum wire as a counter electrode and an ‘Ag/AgOTf, R’ reference electrode (where R represents the IL under study). This reference electrode was prepared by immersing a silver wire (ALS, Tokyo, Japan) in the ionic liquid of interest containing 10 mM AgOTf and separated by a porous glass frit from the test solution. However, to minimise uncertainties related to potential variation caused by the use of reference electrodes containing different ILs and changes in liquid junction potentials between the test solution and the reference electrode and to ease the comparison between different ILs [4,33,34], each reference electrode was calibrated against Fc (Table 2), and the peak potential or *E*_m_ data [*E*_m_ = 1/2($E_{p}^{ox}$ + $E_{p}^{red}$)] of **1** obtained in each IL system reported vs. Fc^0/+^ redox system. It should be noted that the *E*_m_ of ferrocene is affected by the nature of the IL used, and care should be practised to compare the redox potentials [4,34,35]. Prior to each experiment, the working electrode was polished with 0.3 mm alumina (Buehler, Lake Bluff, IL, USA) on a clean polishing cloth (Buehler), sequentially rinsed with distilled water and acetone, and then dried with lint-free tissue paper.

## 4. Conclusions

The present investigation reveals the wide range of electrochemical environments available with ILs and how the media influence the electrochemical reversibility for the oxidation of **1**.

The reactivity of the four anions employed in this work follows the order [NTf_2_]^–^ < [BF_4_]^–^ < [OTf]^–^ < [PF_6_]^–^, indicating that the initial classification of these anions into a “neutral” category is incorrect, as all of them showed a different level of reactivity, and then different Lewis base properties, towards electron-deficient **1^+^**. For example, when [NTf_2_]^–^ is used, the oxidation of **1** showed good electrochemical reversibility, even at low scan rates or when the IL was used as the supporting electrolyte in CH_2_Cl_2_, whereas when [bmim][PF_6_] was used, the oxidation process was irreversible. On the other hand, in tetrafluoroborate, and trifluoromethanesulfonate-based IL, the oxidation of **1** shows different levels of electrochemical reversibility with a marked sign of anion attack to **1^+^**. These results suggest that **1^+^** is more sensitive than the previously used [(Cp)Fe(CO)_2_]_2_ [3], towards small differences in Lewis basic properties of the IL anions.

With many ILs commercially available and several others readily synthesised in a research laboratory, it should be possible, by the informed choice of the specific anions used, to tune the properties of IL-based electrochemical media for a host of applications, including those requiring exceptionally non-coordinating conditions.

## Figures and Tables

**Table 1:** Cyclic Voltammetric data obtained for oxidation of 5 mM **1** in ILs.

IL	Scan Rate V s^−1^	$I_{p}^{ox}$ μA	∆E_p_ mV	*E*_m_ V	*E*_p−_*E*_p/2_ mV	IpredIpox *^c^*	10^7^ *D ^d^* cm^2^ s^−1^	*k*_1_*^f^* s^−1^
[bmim][PF_6_]	0.1	1.79	-	0.891 *^a^*	91	-	-	1.8 × 10^4^
	0.2	2.70	-			-		
	0.4	3.50	-			-		
[bmim][BF_4_]	0.1	1.42	67	0.831 *^b^*	68	0.76	1.2 *^d^*	18
	0.2	1.90	75			0.76		
	0.4	2.68	75			0.82		
[emim][NTf_2_]	0.1	2.02	72	0.838 *^b^*	64	0.75	3.5 *^d^*	0.45
	0.2	2.92	75			0.83		
	0.4	4.27	84			0.87		
[bmpyr][NTf_2_]	0.1	1.26	71	0.840	62	0.83	1.6 *^d^*	0.45
	0.2	1.79	76			0.89		
	0.4	2.46	88			0.92		
[emim][OTf]	0.1	2.42	-	0.869 *^a^*	77	-	3.5 *^e^*	8 × 10^3^
	0.2	3.08	-			0.57		
	0.4	4.08	-			0.63		

*^a^* Peak potential ($E_{p}^{ox}$) for oxidation of **1** vs. *E*_m_ of Fc^0/+^; *^b^* Midpoint potential (*E*_m_) for oxidation of **1** vs. *E*_m_ of Fc^0/+^, where Em was calculated from the average of the oxidation ($E_{p}^{ox}$) and reduction ($E_{p}^{red}$ ) peak potentials and is approximately equal to the reversible formal potential ($E_{f}^{0}$). *^c^* Values obtained with the equation $I_{p}^{red}/I_{p}^{ox}={\left(I_{p}^{red}\right)}_{0}/I_{p}^{ox}+0.485\;{\left(I_{f}\right)}_{0}/I_{p}^{ox}+0.086$, were ${\left(I_{f}\right)}_{0}$ is the current measured at the switching potential. *^d^ D* = Diffusion coefficient of **1** calculated from chronocoulometry and cyclic voltammetry. *^e^ D* = Diffusion coefficient of **1** calculated by cyclic voltammetry simulation. *^f^* Pseudo-first order kinetic rate constant obtained by the best fit of experimental and simulated voltammograms.

**Table 2:** Mid-point potential of Fc^0/+^ against different Ag/AgOTf, R reference electrode.

Reference Electrode	*E_m_* (V)
Ag/AgOTf, [emim][OTf]	−0.250
Ag/AgOTf, [bmim][PF_6_]	0.043
Ag/AgOTf, [bmim][BF_4_]	−0.583
Ag/AgOTf, [bmpyr][NTf_2_]	−0.391
Ag/AgOTf, [emim][ NTf_2_]	−0.374
